# A case of outpatient treatment in a 58-year-old woman with hoarding disorder and hallucinations.

**DOI:** 10.1192/j.eurpsy.2023.522

**Published:** 2023-07-19

**Authors:** A. Gonzalez-Mota, A. Gonzalez-Gil, M. T. Basanta-Patiño, M. Colomer-Sagaste

**Affiliations:** ^1^Psychiatry, University Hospital Complex of Salamanca; ^2^Institute of Biomedicine of Salamanca (IBSAL), Salamanca; ^3^Psychology, University Hospital Miguel Servet; ^4^Psychology, Royo Villanova Hospital, Zaragoza; ^5^Psychology, Maternal and Child Insular Hospital, University Hospital of Gran Canaria, Las Palmas, Canary Islands, Spain

## Abstract

**Introduction:**

In hoarding disorder the patient has a strong tendency to collect and accumulate objects with or without value and great difficulty in destroying them.

In this case, a 58-year-old woman diagnosed with a hoarding disorder 5 years ago, came to a psychiatry clinic due to frequent auditory hallucinations related to episodes of acute stress. She received treatment in an outpatient mental health unit which consisted of psychopharmaceuticals and cognitive behavioural therapy. The patient achieved a partial remission of the hallucinations and a clinical improvement of the accumulation symptoms.

**Objectives:**

The main objective of this study is to describe the psychiatric and psychological treatment of this patient. We also performed a review of the available literature on comorbidity of the symptoms of Diogenes syndrome and psychotic symptoms.

**Methods:**

A close follow-up of the psychopathology of this patient was carried out and we did a database search in PubMed to document the case, with the keywords: “hoarding disorder”, “psychotic disorder”, “comorbidity”, “hallucination”, with the inclusion criteria: In the last ten years, Spanish and English language.

**Results:**

The patient, who was being treated with sertraline 100 mg, started treatment with olanzapine 10 mg and with a psychotherapeutic plan with different objectives: stabilization of symptoms, reduction of hoarding behaviours, letting go of objects, as well as coping with stressful situations. Cognitive behavioural techniques such as psychoeducation, exposure with response prevention and cognitive therapy were included in the psychological treatment.

After one year of treatment the hallucinatory symptoms have remitted and the patient’s daily functioning has improved. The most resistant symptoms are those of accumulation that are slowly decreasing but the patient has stopped collecting objects from the street.

**Conclusions:**

More studies of the treatment of hoarding disorder and more investigation of its possible comorbidities are needed.

**Disclosure of Interest:**

None Declared

